# *In vitro* study of low intensity ultrasound combined with different doses of PDT: Effects on C6 glioma cells

**DOI:** 10.3892/ol.2012.1060

**Published:** 2012-12-04

**Authors:** JIAN-HUA LI, ZHI-QIANG CHEN, ZHENG HUANG, QI ZHAN, FU-BIN REN, JING-YE LIU, WU YUE, ZHI WANG

**Affiliations:** 1 Department of Neurosurgery, The Fourth College Hospital of Harbin Medical University, Harbin, P.R. China;; 2 Department of Radiation Oncology, Colorado University of Health Sciences Center, Denver, CO, USA

**Keywords:** sonodynamic and photodynamic therapy, hematoporphyrin monomethyl ether, apoptosis, reactive oxygen species, C6 glioma cells

## Abstract

The aim of this study was to study the effects of killing C6 glioma cells induced by hematoporphyrin monomethyl ether (HMME)-mediated sonodynamic therapy combined with photodynamic therapy (SPDT). In the SPDT group, the cells were treated with sonication at an intensity of 0.5 W/cm^2^ and a frequency of 1 MHz, followed by different doses of light irradiation. The growth inhibition rate following treatment was determined by MTT assay. The apoptotic rate was examined by a flow cytometry. Cleavage of caspase 3, 8 and 9 was investigated by immunoblotting. Reactive oxygen species (ROS) were measured by a fluorescence microplate reader. The effect of SPDT on the glioma cells was also studied in the absence or presence of various ROS scavengers. The growth inhibition rate of C6 glioma cells treated with SPDT was significantly higher compared with sonodynamic therapy (SDT) or photodynamic therapy (PDT) alone at light doses <200 J/cm^2^. The growth inhibition rate of C6 glioma cells treated with SPDT did not rise significantly when the light dose increased to >120 J/cm^2^. The apoptosis rate was the highest in the SPDT group, when the light dose was at 80 J/cm^2^. A greater amount of ROS were generated in the SPDT group than in the groups treated with SDT or PDT alone. The addition of NaN_3_ or mannitol resulted in a decrease in the growth inhibition rate with SPDT. In conclusion, our data indicate that SPDT powerfully kills C6 glioma cells *in vitro* through the synergistic effects of SDT and PDT. The pathway of PDT inducing C6 glioma cell apoptosis includes both the mitochondrial and death receptor pathways. Furthermore, ROS may play an important role in SPDT.

## Introduction

Photodynamic therapy (PDT) is widely used in the therapy of gliomas at a biological level and in the clinic. PDT-mediated generation of molecular oxygen or reactive oxygen species (ROS) is assumed to be the main mechanism of cell death (1), and plays an important role in killing glioma cells. However, PDT has certain disadvantages; for example, its initial killing effect is not satisfactory. The effective depth of PDT is limited, as lasers are unable to penetrate and reach deep tissues to activate the photosensitizer.

Ultrasound has an appropriate tissue attenuation coefficient for penetrating and reaching deep-seated tissues while maintaining the ability to focus energy onto a small volume. This unique advantage makes it more useful for noninvasive treatment of deep-seated tumors when compared with electromagnetic modalities, such as laser beams (2–4). These techniques may be applied to the treatment of numerous types of cancer. However, a study has reported that the delayed killing effect is unsatisfactory in sonodynamic therapy (SDT), and advise that an alternative therapeutic method should be combined with PDT (5).

It is of note that certain sensitizers can be activated photochemically as well as sonochemically (6–8), such as porphyrin sonosensitizers (3,4), as well as others (9–11). PDT combined with SDT (SPDT) is a new cancer therapy. The theory of the therapy is that by activating these congenerous sensitizers with light and sound, more cytotoxicity is generated to kill tumor cells. A number of scholars have reported the effects of killing tumor cells by SPDT; for example, Kessel *et al*(12) demonstrated that photodamage following exposure to ultra-sound decreased the viability of murine leukemia L1210 cells which had survived ultrasonic treatment. Jin *et al*(13) studied SPDT for improving tumoricidal effects in a transplantable mouse squamous cell carcinoma (SCC) model, and showed that combination therapy induced tumor necrosis 2–3 times as deep as with either of the single modalities, concluding that SPDT may be useful in the treatment of non-superficial or nodular tumors. Kolarova *et al*(14) demonstrated that more ROS were generated using SPDT than using a monotherapy of PDT or SDT.

Nonetheless, research in this domain is rare, and to date, the effects of SPDT and the biological mechanisms by which it kills different tumor cell lines are undefined. Therefore, in the present study, we investigated the mechanisms by which PDT combined with SDT activates hematoporphyrin monomethyl ether (HMME) to kill C6 rat gliomas cells. We also studied the interactions between the two modalities.

## Materials and methods

### Cell cultivation

Rat glioma C6 cells were obtained from the Neurosurgery Institution of Harbin Medical University, China. C6 cells were maintained as monolayers in Roswell Park Memorial Institute (RPMI)-1640 medium (Hyclone Lab, Logan, UT, USA) at 37°C with 5% CO_2_ in a humidified incubator (Nuaire, Plymouth, MN, USA). Cells in the exponential phase of growth were used for all the following experiments. The study was approved by the Ethics Committee of Haerbin Medical University, Harbin, China.

### Sonodynamic and photodynamic treatment

All operations were carried out at 37°C. Exponentially growing cells were collected by centrifugation, resuspended (5×10^6^ cells/ml) in serum-free RPMI-1640 medium and incubated with HMME for 4 h. In the experiments, a multifunction physical therapy ultrasound device (Tianshi Technologies Ltd. Co, Beijing, China) was used to generate ultrasound at 1 MHz in cells in the presence of 10 *μ*g/ml HMME. Ultrasonic intensities (0.5 W/cm^2^) were measured by a stainless steel ball radiometer (diameter, 0.32 cm) (15). Further details are provided in a previous study by Li *et al*(16). Following this, cells were irradiated with a certain dose of light. For the irradiation, the wavelength was limited to 630 nm by an interference filter. The light was set to 100 mW/cm^2^ for subsequent irradiation up to different doses of light using a semiconductor diode laser (Diomed 630, Andover, MA, USA).

The samples were divided into different groups. In the control group, the cells were not treated with HMME, nor with ultrasound or light irradiation. In the HMME group, the cells were treated with HMME but not with ultrasound or light irradiation. In the ultrasound and light irradiation groups, the cells were treated with ultrasound or light irradiation alone. In the SDT group, the cells were treated with both HMME (10 *μ*g/ml) and ultrasound. In the PDT group, the cells were treated with both HMME (10 *μ*g/ml) and light irradiation. In the SPDT group, the cells were treated with HMME (10 *μ*g/ml) and sonication at an intensity of 0.5 W/cm^2^, followed by exposure of 90 sec; subsequently, cells were irradiated with different doses of laser light.

### Cell survival assay

After the treatment cells were washed, re-suspended in RPMI-1640 medium and subjected to MTT [3-(4,5-dimethylthiazol-2-yl)-2,5-diphenyltetrazolium bromide] assay. MTT (20 *μ*l; 5 mg/ml) was added. Four hours later, 100 *μ*l DMSO was added to each well to dissolve the resulting formazan crystals. Absorbance was read at 490 nm using an enzyme-linked immunosorbent assay reader (SpectraMax; Molecular Devices, Sunnyvale, CA, USA). Survival rate was calculated as: Inhibition rate (%) = (1 − OD_treatment group_ / OD_control group_) × 100, where OD indicates optical density.

### Examination of apoptosis or necrosis

Following treatment, cells were re-incubated for up to 4 h in the dark and washed twice with phosphate-buffered saline (PBS). After adjusting the cell density to 1×10^6^ cells/ml, 100 *μ*l cell suspension was transferred to a culture tube and mixed with 5 *μ*l Annexin V-FITC (fluorescein isothiocyanate; BD, Franklin Lakes, NJ, USA) and 5 *μ*l propidium iodide (PI; BD). The mixture was gently vortexed and incubated at room temperature (25°C) in the dark for 15 min. After adding 400 *μ*l binding buffer, the apoptotic rate was analyzed using a flow cytometer (BD).

### Western blot analysis of caspase 3, 8 and 9 activation

Following treatment, cells were re-incubated for up to 4 h in the dark. To examine caspase 3, 8 and 9 activation, cells of different groups were separately washed, collected and homogenized in a lysis buffer (10 mM Tris-HCl, pH 8, 0.32 mM sucrose, 5 mM EDTA, 2 mM DTT, 1 mM phenylmethyl sulfonylfluoride and 1% Triton X-100) and then centrifuged. Proteins in different groups were separately electrophoresed on SDS polyacrylamide gel (12%), the gel-separated proteins were transferred to nitropure nitrocellulose membranes (Santa Cruz Biotechnology, Inc., Santa Cruz, CA, USA), and the membranes were probed overnight at 4°C with primary antibodies. Each of the targeted proteins was immunostained by distinct antibodies. The antibodies presented were: anti-actin, anti-cleavage caspase 3, 8 and 9 (Santa Cruz Biotechnology, Inc.). After probing, the membranes were washed three times and then incubated for 1 h at room temperature with the respective alkaline phosphatase-conjugated secondary antibodies (Sigma, St. Louis, MO, USA) before being visualized using a chemiluminescence detection kit (Sigma).

### Measurement of ROS generation

2’,7’-Dichlorofluorescein diacetate (DCFH-DA; Beyotime Institute of Biotechnology, Shanghai, China) was used to detect SPDT-mediated ROS production. DCFH-DA was added to the cell suspension at a final concentration of 10 *μ*mol/l and incubated at 37°C in the dark for 30 min. Fluorescent dichlorofluorescein generated from the oxidation of DCFH-DA was measured by a fluorescence microplate reader (FLx800; BioTek, Winooski, VT, USA).

### Cell survival assay following addition of different ROS scavengers

To confirm the involvement of ROS in SPDT, the above procedures (SDT) were repeated in the presence of either 100 *μ*g/ml final concentration of sodium azide (NaN_3_) or 100 *μ*g/ml final concentration of mannitol (C_6_H_14_O_6_). Subsequently, cells were subjected to an MTT assay.

### Statistical analysis

Statistical evaluation was performed with a t-test using analytical software tools from SPSS. Data are presented as the mean values ± standard error of the mean (SE). P<0.05 was considered to indicate a statistically significant result.

## Results

### Inhibition of cell growth with SPDT compared with PDT or SDT

**Figure 1.**
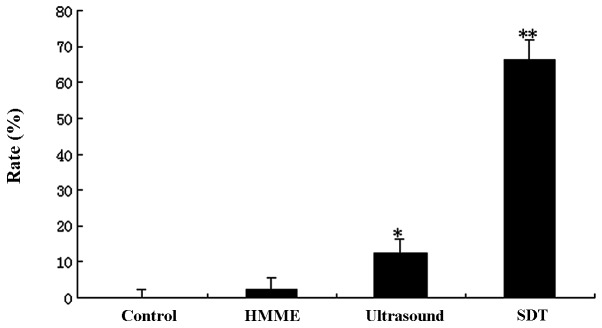
Growth inhibition rate of rat C6 glioma cells measured by MTT assay following sonodynamic therapy (SDT) treatment. Data represent mean ± SE (n=6). ^**^P<0.01 vs. control, ^*^P<0.05 vs. control. HMME, hematoporphyrin monomethyl ether.

**Figure 2.**
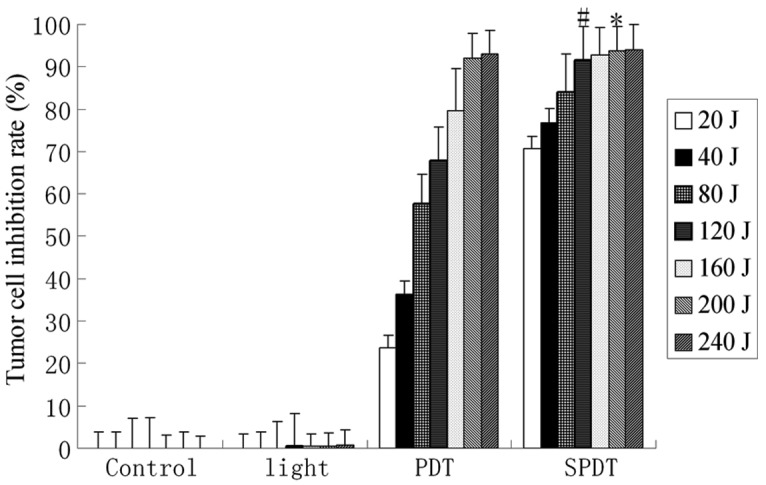
Growth inhibition rate of rat C6 glioma cells in different groups were measured by MTT assay. Data represents mean ± SE (n=6). ^*^P<0.05, SPDT vs. PDT (light irradiation dose <200 J/cm^2^). ^#^P<0.05, SPDT growth inhibition rate (light irradiation dose, 120 J/cm^2^) vs. other groups of SPDT (light irradiation dose <120 J/cm^2^). SDT, sonodynamic therapy; PDT, photodynamic therapy.

The growth inhibition rate of C6 glioma cells was determined by MTT assay in SDT combined with different doses of light irradiation (20–240 J/cm^2^). Although PDT or SDT alone also inhibited cell growth, the inhibition rate in the SPDT group was significantly higher than that in the SDT or PDT groups at certain doses (light dose <200 J/cm^2^; P<0.05). When the light dose was >200 J/cm^2^, the inhibition rate was not significantly different from that in the PDT group (P>0.05). However, in the SPDT groups, when the light dose was >120 J/cm^2^, the inhibition rate was not significantly increased with increasing light dose (P>0.05). Thus, our data indicates that HMME-mediated SPDT induced a synergetic killing effect on C6 cells at specific light doses (Figs. 1 and 2). A similar killing effect was achieved by SPDT with lower doses of light compared with PDT alone with higher doses of light.

### SDT-induced apoptosis

**Figure 3.**
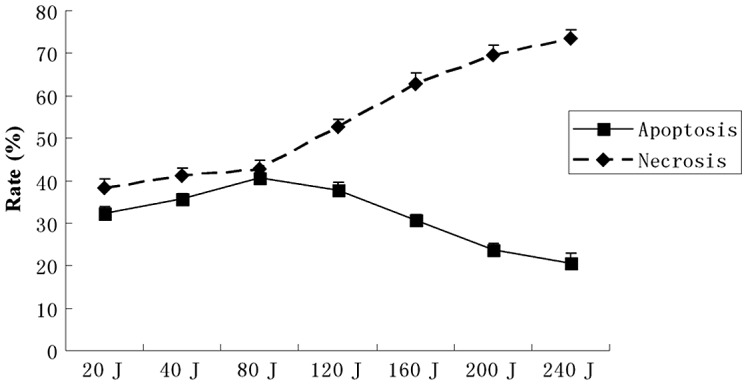
Sonodynamic therapy (SDT) combined with different doses of light irradiation (20–240 J/cm^2^) induced C6 cell apoptosis or necrosis, evaluated by flow cytometry. Data represent mean ± SE (n=6).

**Figure 4.**
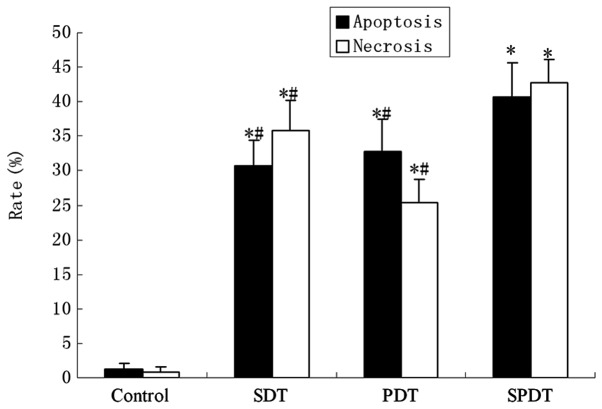
Apoptosis and necrosis measured in rat C6 glioma cells 4 hours after different treatment as measured by flow cytometry of Annexin V-PI treated cells. Data represent mean ± SE (n=6). ^*^P<0.05 vs control;. ^#^P<0.05 vs. SPDT. SDT, sonodynamic therapy; PDT, photodynamic therapy; SPDT, sonodynamic therapy combined with photodynamic therapy.

The flow cytometry assay showed marked changes in the cell profile following SPDT. Using flow cytometry, we evaluated the effect of combining SDT with different doses of light irradiation (20–240 J/cm^2^) on apoptosis or necrosis in C6 cells. In the SPDT group, the cell apoptosis rate rose with the increasing light dose when the light irradiation dose was <80 J/cm^2^. Conversely, the cell apoptosis rate reduced as the light dose increased when the light dose was >80 J/cm^2^; however, the necrosis rate increased notably when the light dose was >80 J/cm^2^. The results suggested that SPDT induced the highest level of cell apoptosis when SDT was combined with a light irradiation dose of 80 J/cm^2^ (Fig. 3). The apoptotic rate was ∼40.62±5.01% with a light irradiation dose of 80 J/cm^2^ in SPDT. The apoptosis rate of C6 glioma cells in the SPDT group was the highest among all groups (P<0.05), while the SDT and PDT groups were higher compared with the control (P<0.01), but lower than the SPDT group (P<0.05). HMME-mediated SPDT also increased the apoptotic rate of C6 cells in certain conditions (Fig. 4).

### SPDT induces caspase 3, 8 and 9 activation

**Figure 5.**
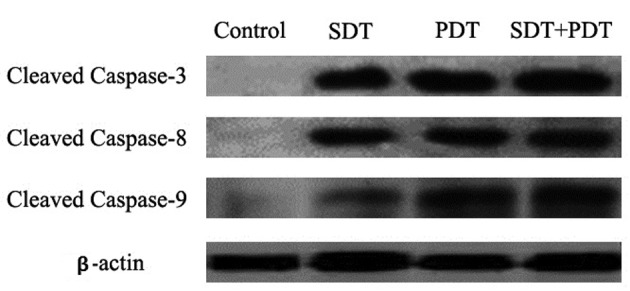
Caspase 3, 8 and 9 cleavage in C6 cells following various treatments. Representative western blots for the expression of caspase 3, 8 and 9 cleavage in different groups. SDT, sonodynamic therapy; PDT, photodynamic therapy; SPDT, sonodynamic therapy combined with photodynamic therapy.

As shown by the western blot analysis of cytosolic extracts, HMME-SPDT induced caspase 3, 8 and 9 cleavage with a light irradiation dose of 80 J/cm^2^ in SPDT. Band intensity quantitation measurements showed that the activation rate of caspase 3, 8 and 9 was markedly higher than that in the SDT or PDT groups alone (Fig. 5).

### Effect of ROS on SDT-induced cell killing

**Figure 6.**
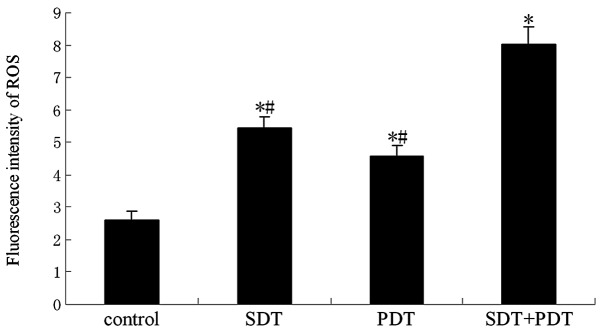
Reactive oxygen species (ROS) production as indicated by DCFH fluorescence in different groups. Data are mean ± SE (n=6). ^*^P<0.05 vs. control; ^#^P<0.05 vs. SPDT. SDT, sonodynamic therapy; PDT, photodynamic therapy; SPDT, sonodynamic therapy combined with photodynamic therapy.

**Figure 7.**
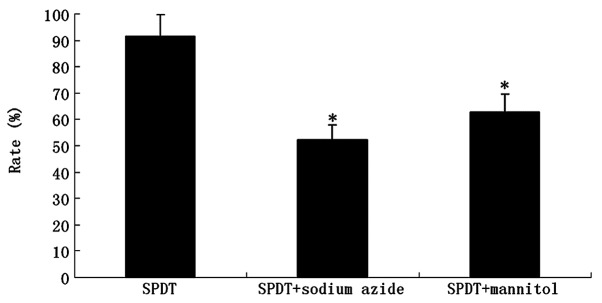
Effect of scavengers on the SPDT-induced cell death (mean ± SE, n=6). Growth inhibition rate of rat C6 glioma cells in different groups were measured by MTT assay. ^*^P<0.05, compared with SPDT group. SPDT, sonodynamic therapy combined with photodynamic therapy.

To demonstrate the effect of ROS in SPDT-induced cell killing, ROS production was confirmed by the oxidation of DCFH-DA. The results showed that the synergistic effect of SDT and PDT generated more ROS than SDT or PDT alone at a light irradiation dose of 80 J/cm^2^ in SPDT (P<0.01; Fig. 6). HMME-mediated SPDT killing of glioma cells was studied in the absence or presence of various ROS scavengers (e.g., NaN_3_ and mannitol). The presence of NaN_3_ significantly reduced the inhibition rate of HMME-mediated SPDT. At the level of 100 *μ*g/ml, the singlet oxygen scavenger caused a reduction of almost 39% (P<0.01). The presence of mannitol also reduced HMME-mediated SPDT killing by ∼29% (P<0.05; Fig. 7).

## Discussion

SPDT is a new, combined therapy for treating cancer. The basis of the therapy is to administer a small amount of sensitizer, which is selectively taken up by cancer cells, and then expose the body to light and sound to activate these sensitizers (17–19). These techniques are known to have a cancer-killing effect and are applicable to a wide range of cancers, but the effect of killing malignant glioma cells is unknown. We therefore used light and sound to activate the photosensitizer, a hematoporphyrin derivative (HMME), and examined its effectiveness and mechanisms of killing glioma cells.

For evaluating the effects of HMME-mediated SPDT, we first determined the growth inhibition rate of C6 glioma cells in the SPDT group compared with other groups. Our results indicated that HMME-mediated SPDT induced a synergetic killing effect on C6 cells when the light dose was <200 J/cm^2^ (Figs. 1 and 2). SPDT at a lower light dose achieved a better synergetic killing effect than the monotherapies. In the SPDT groups, when the light dose was greater than 120 J/cm^2^, the inhibition rate was not significantly increased with increasing light dose (P>0.05). This indicated that SPDT had a sufficient killing effect when ultrasound generated 1 MHz frequency, 0.5 W/cm^2^ intensity and light irradiation up to 120 J/cm^2^. SPDT effectively killed C6 cells with lower doses of light compared with PDT alone. Hence, we determined that HMME-mediated SPDT was a good method for killing C6 gliomas.

Apoptosis is one of major modes of tumor cell death (20). In this experiment, we used light and sound to activate the photosensitizer HMME and examined its effectiveness and the mechanisms by which it induces C6 glioma cell apoptosis. Cell apoptosis was observed in HMME-mediated SPDT *in vitro*. In our experiment, we revealed that the apoptosis rate of C6 cells significantly increased when the light irradiation dose was lower than 80 J/cm^2^; however, when the light irradiation dose was above 80 J/cm^2^, the necrosis rate of C6 cells significantly increased instead of apoptosis. Experimental results indicated that HMME-mediated SPDT induced the highest rate of C6 glioma cell apoptosis at a light dose of 80 J/cm^2^. These results indicated that SDT and PDT had a synergic effect on inducing tumor cell apoptosis at a certain light dose. It suggests that SPDT with low doses of light may induce major C6 cell apoptosis; but in contrast it mainly results necrosis with high doses of light.

As we know, apoptosis is often initiated by either an extrinsic (activated caspase 8) or an intrinsic pathway (activated caspase 9). The extrinsic pathway functions can directly activate caspase 8 through the death receptors on the cell surface; however, the intrinsic pathway regulates the activation of caspase 9, and subsequently the activation of caspase 3. In the present study, we attempted to indentify the intrinsic or extrinsic apoptosis pathways using an SPDT *in vitro* model. Our experiment also demonstrated that HMME-mediated SPDT magnified the release of cleaved caspase 3, 8 and 9 in cytoplasm. The changes in the SDT or PDT group were not as apparent as those in the SPDT group. These results showed that both the mitochondrial and death receptor pathway may be two channels by which SPDT induces C6 glioma cell apoptosis.

Certain studies have demonstrated that ROS were generated following by SDT or PDT (11,21). Oxidative stress has been reported to initiate the killing effect in SDT or PDT (22–24). In our experiment, we also detected that SPDT increased ROS levels *in vitro* (Fig. 6). Although SDT or PDT alone increased the generation of ROS, ROS generation in these groups was much lower than that in the SPDT group. The presence of NaN_3_ or mannitol significantly reduced the inhibition rate of HMME-mediated SPDT. It suggested that increased ROS generation was the key factor to killing C6 cells. These results are similar to those found in other reports (12,13).

In summary, these results suggest that HMME-mediated SPDT may be a promising method for the treatment of glioma. Combined SDT with PDT at various conditions may cause different biological effects.
